# Adult Blaschkitis With Lichenoid Features and Blood Eosinophilia

**DOI:** 10.7759/cureus.16846

**Published:** 2021-08-03

**Authors:** Amal Al-Balbeesi

**Affiliations:** 1 Dermatology, King Khalid University Hospital, Riyadh, SAU

**Keywords:** blaschkitis, mosaic, lichen striatus, eosinophilia, allergic response

## Abstract

There have been many reports of congenital and acquired dermatoses that trail Blaschko lines. Lichen striatus is representative of an acquired cutaneous linear inflammatory dermatosis running along the lines of Blaschko, characterized histopathologically by the predominance of lichenoid infiltration. Adult blaschkitis, however, is considered under the same disease spectrum as lichen striatus and is characterized by a spongiotic reaction pattern. Few differences have been recognized between lichen striatus and adult blaschkitis such as age of onset, triggers, distribution, histopathology, and response to treatment.

A case of male patient with adult blaschkitis presenting as unilateral asymptomatic erythematous edematous papules, papulovesicles, and plaques over Blaschko’s lines, in which skin biopsy revealed coexistent pathological features of lichen striatus and adult blaschkitis along with blood eosinophilia, has been presented here. This may add to the cumulative evidence on the pathogenesis of adult blaschkitis as an allergic reaction to an unknown inciting allergen.

Evidence to consider adult blaschkitis as part of the lichen striatus spectrum is growing. Triggers for such skin reactions remain diverse. This case presentation suggests that adult blaschkitis could be triggered by an allergic response evidenced by the blood eosinophilia when other causes of eosinophilia are excluded.

## Introduction

Blaschko’s lines (BL) are considered systematized cutaneous developmental patterns during embryogenesis that are different from vascular, neural, or lymphatic pathways. The BL were described and drawn by Alfred Blaschko, a German dermatologist, in 1858-1922 [1,2].

There have been many reports of congenital and acquired dermatosis that trail BL. Congenital dermatoses located on BL are incontinentia pigmenti, epidermal nevus, linear nevus sebaceous, and focal dermal hypoplasia (Goltz syndrome) [3-5].

Blaschkolinear acquired inflammatory skin eruption (BLAISE) is a cluster of disorders consisting of various inflammatory skin diseases including lichen striatus (LS), adult blaschkitis (AB), lichen planus (LP), psoriasis, lichen nitidus, atopic dermatitis, lupus erythematosus, and graft-versus-host disease [4]. We report a case of AB with coexisting pathological features of LS and transient blood eosinophilia, which resolved with the clearing of skin lesions, suggesting an allergic reaction as a possible trigger for the blaschkitis or an associated feature of the skin presentation since other causes of eosinophilia were excluded.

## Case presentation

**Figure 1:**
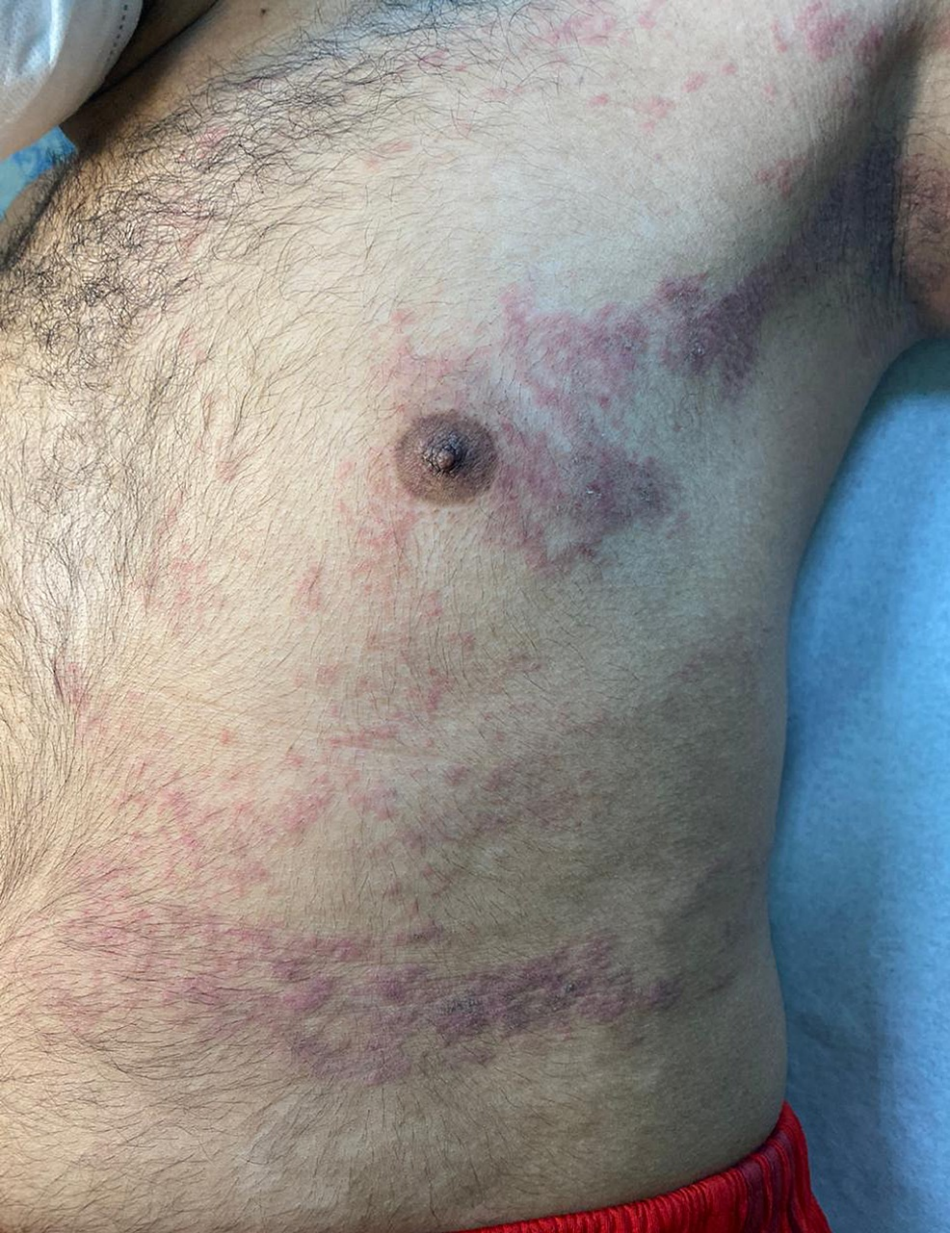
Spread of deep-seated vesicles on the arm, trunk, and lower extremity.

A 19-year-old Yemeni male patient (skin type 5) presented to dermatology with a two-week history of a minimally itchy eruption that involved the left side of his trunk, axilla, upper, and lower extremities. Lesions started as deep-seated vesicles on the palm and then spread centripetally to involve the arm, trunk, and lower extremity, as seen in Figure 1.

**Figure 2:**
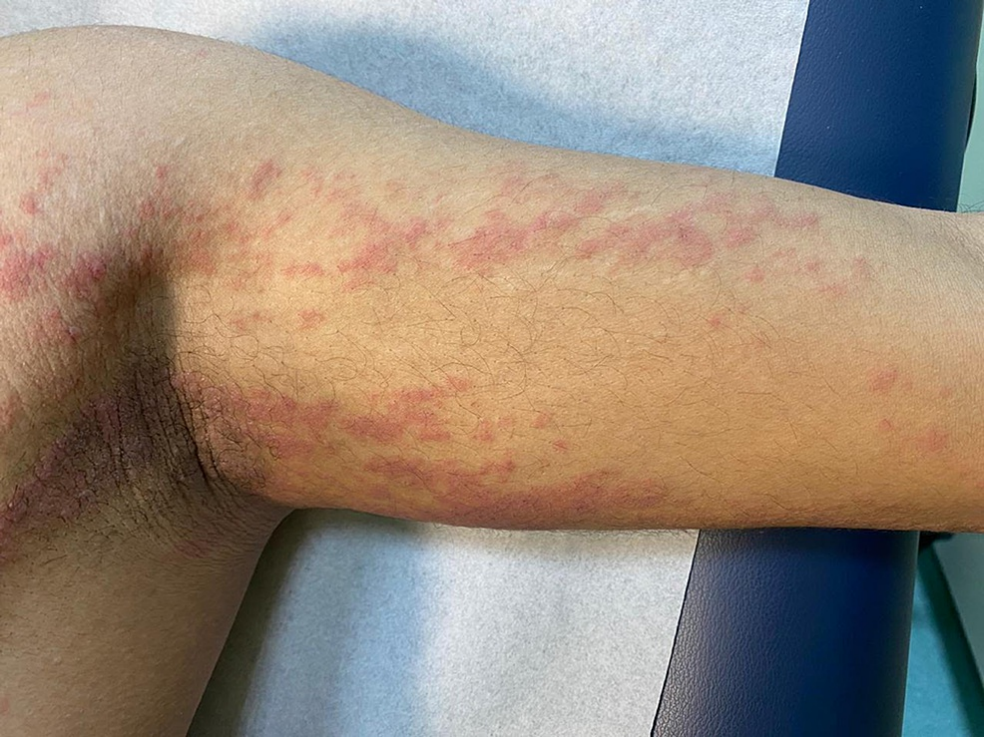
Unilateral erythematous edematous papules, plaques, and papulo-vesicles over the left side of the body.

There was no personal history of atopy, insect bite, drugs, or recent infection, nor a family history of similar presentation. On examination, unilateral erythematous edematous papules, plaques, and papulo-vesicles over the left side of the body were observed, as seen in Figure 2.

The lesions were following BL as whorls and interrupted lines. Nails and mucous membranes were spared. Complete blood count (CBC) showed hemoglobin and white blood cell count to be within normal limits and eosinophilia of 10.6%. Additionally, renal and liver functions were normal, hepatitis B screening was negative, and antinuclear antibody and stool analysis were negative.

**Figure 3:**
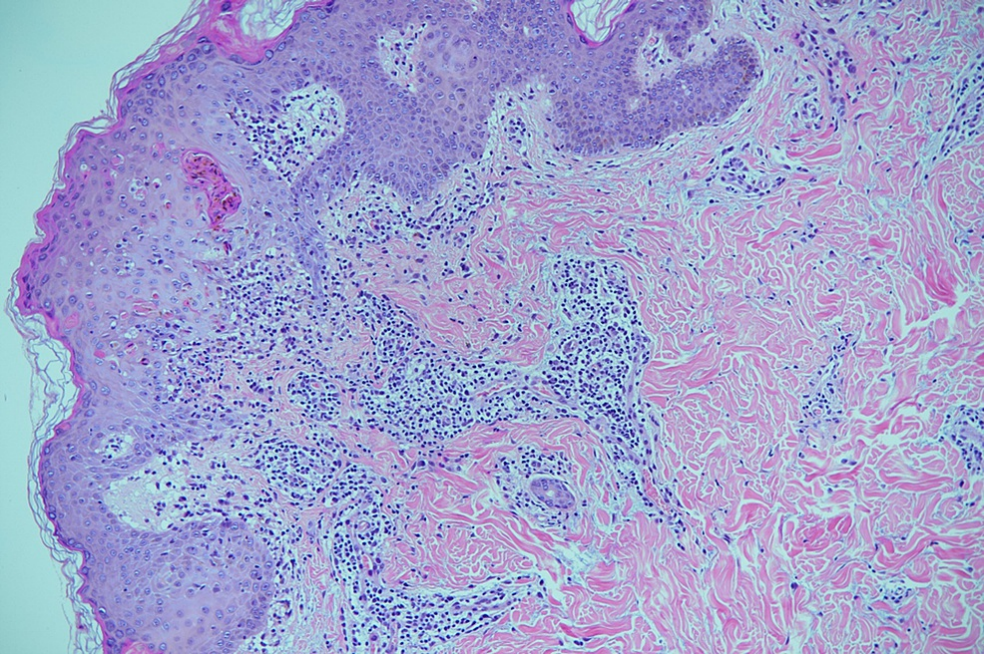
Patterns that were consistent with spongiotic and lichenoid reactions with eosinophils.

**Figure 4:**
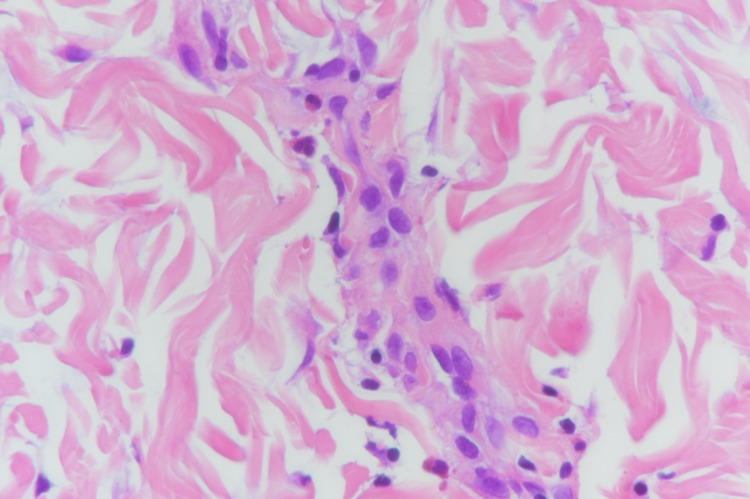
Scattered eosinophils (seven eosinophils per 10 high-power fields).

Biopsy showed parakeratosis, epidermal spongiosis, and mild acanthosis with several necrotic keratinocytes mainly at the lower levels of the epidermis. In the dermis, foci of lichenoid infiltrate at the dermo-epidermal junction were found. Lymphocytic infiltrate was also noted around the eccrine ducts. Additionally, scattered eosinophils (seven eosinophils per 10 high-power fields), some extravasated red blood cells, and no evidence of vasculitis were observed, as seen in Figure 3, Figure 4.

**Figure 5:**
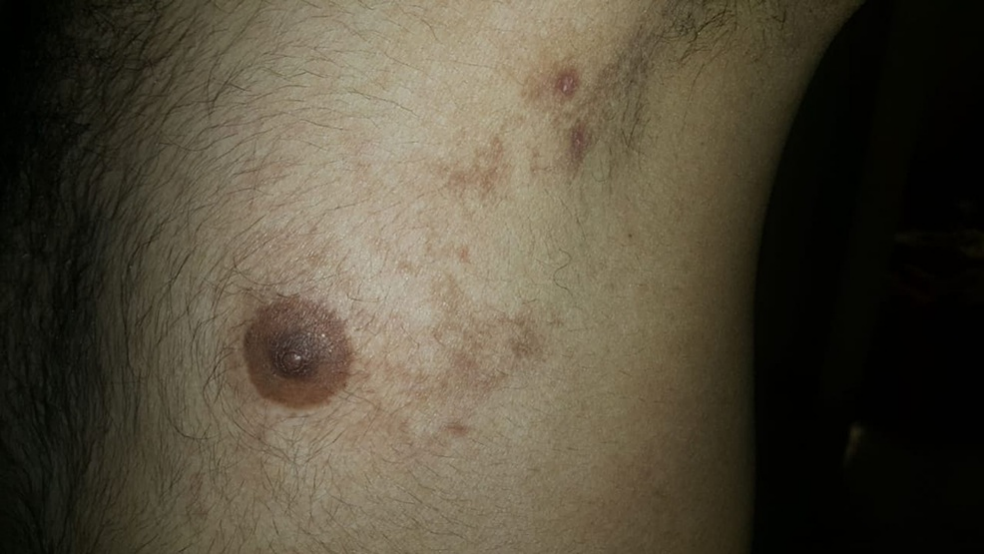
The lesions resolved with post-inflammatory hyperpigmentation spontaneously within two weeks.

Immunofluorescent studies for IgA, IgG, IgM, C3, and fibrinogen were negative. The lesions resolved with post-inflammatory hyperpigmentation spontaneously within two weeks, as shown in Figure 5.

Based on the clinical and histopathological features, the diagnosis of AB with spongiotic dermatitis and the coexistent lichenoid infiltrate with eosinophils was considered. The patient was prescribed topical mometasone cream 0.1% and a poor compliance was reported but was nonetheless satisfied with the outcome. CBC repeated after 12 weeks revealed no eosinophilia and there was no recurrence of lesions.

## Discussion

AB is one of the disorders comprising BLAISE along with many others including LS, LP, psoriasis, lichen nitidus, atopic dermatitis, lupus erythematosus, and graft-versus-host disease [4]. AB and LS are still considered under the same disease spectrum; however, few differences have been recognized, such as age of onset, distribution, histopathology, and response to treatment[6-9] (Table 1).

**Table 1:** Differences between adult blaschkitis and lichen striatus [6-24]. +++, frequently reported; +, less commonly reported. BL, Blaschko’s lines

Features	Adult Blaschkitis	Lichen Striatus
Age in years	18-55	5-15
Distribution	Multiple sites including extremities and trunk	Unilateral single site (extremities)
Clinical	Papulo-vesicles over multiple BL	Erythematous-brown discrete and coalescing papules over BL
Symptoms	Itchy	Sometimes itchy
Time of resolution	Few weeks	One year
Triggers	Pregnancy Infections, vaccination, drugs, trauma insect bites, stem cell transplant	Infection, sunburn, contact allergens
Histopathology		
Spongiosis	+++	+++
Lichenoid infiltrate	+	+++
Treatment		
Response to treatment	Favorable	Less favorable
Spontaneous resolution	+++	+++
Recurrence	Common	Rare

LS is representative of acquired cutaneous linear inflammatory dermatosis running along the BL, usually in a unilateral fashion and mainly over extremities[6-9]. LS primarily occurs in children between 5 and 15 years of age. Involvement of the nail in LS is rare and may occur before, concurrently, or after the skin lesion [25]. The affected nail may present as pitting, longitudinal ridging, fissuring, splitting, fraying, striate or punctate leukonychia, overcurving, thinning or thickening of the nail plate, irregular transverse grooves, onycholysis, subungual hyperkeratosis, and nail loss. The presentations may only include the lateral or medial portion of the nail plate of only one digit [25]. The case series reported by Lunge et al. showed resolution of LS lesions within three months of tazarotene use with minimal discomfort due to irritation [26]. Topical steroids had been used for itchy LS lesions with inconsistent results [26]. However, calcineurin inhibitors and calcipotriol with topical or intralesional steroids have been used successfully [26]. Spontaneous resolution of LS had been reported within 3-12 months [26]. 

AB affects adults between 15 and 88 years of age and is characterized histopathologically by spongiotic dermatitis primarily as compared to LS, which shares similar pathological features but prominent lichenoid infiltrate [6-10]. Multiple BL are affected over trunk and extremities. Unlike LS, AB is recurrent, a feature that is reported rarely in LS. It is suggested that LS and AB are triggered by many extrinsic stimuli such as pregnancy [11], infection^ ^[12-14], vaccination [15-17], drugs [18-21], trauma [22], insect bite [23], autoimmune phenomena [7,10], and stem cell transplant [24]. These stimuli bring about somatic mutations in keratinocytes resulting in a T-cell immune hypersensitivity response directed against keratinocytes exhibiting mosaicism along BL. 

We report here a case of AB with spongiotic dermatitis and coexistent lichenoid infiltrate with concurrent tissue and blood eosinophilia. Persistent eosinophilia for more than three months can fall into three categories: reactive, clonal, or syndromic such as hypereosinophilic syndrome [27]. Many diseases including allergic conditions such as atopic dermatitis, drugs, infections, autoimmune, and malignancy could lead to eosinophilia [27]. Clinical history is the most important guide to the cause. The lack of any specific finding that points to the cause of eosinophilia in this case including atopic dermatitis made AB the probable cause of eosinophilia. Eosinophils are rare in LP and its variants; however, some cases of hypertrophic LP have eosinophils in the absence of drug history [28]. Hypertrophic LP is to be included in the differential diagnosis of lichenoid dermatitis with eosinophils [28]. Nonetheless, the lack of pruritis, the morphology of the lesions, the short duration, and histopathology are against LP.

We suggest that this patient presentation could be triggered by an allergic reaction to an unknown inciting agent or a true additional feature of AB and to include it in the differential diagnosis of lichenoid dermatitis with eosinophils. Performing a full blood count of such cases may shed light on the etiology and the features of this disease entity.

## Conclusions

In conclusion, AB, a diagnosis subjected to major scrutiny, shares many features with LS and LP. The age of onset, the short duration of the disease, and the histopathological features, all collectively point in favor of AB. Features against LP include the lack of intense pruritis and the clinicopathological features. AB triggers can be diverse; an allergic hypersensitivity reaction could be added to the list and we suggest obtaining eosinophils count to try to understand the triggering factors for such a presentation. Skin eruption resolves spontaneously without treatment with post-inflammatory hyperpigmentation as with our patient and other reported cases. More case reports are needed to understand the triggers of such a presentation to improve our true understanding of the pathogenesis of AB and its associated features.
